# OCT-Angiography: Mydriatic phenylephrine and tropicamide do not influence retinal microvasculature in macula and peripapillary region

**DOI:** 10.1371/journal.pone.0221395

**Published:** 2019-10-17

**Authors:** Bettina Hohberger, Meike Müller, Sami Hosari, Christian Y. Mardin

**Affiliations:** Department of Ophthalmology, Friedrich-Alexander-University of Erlangen-Nürnberg, Erlangen, Germany; University of Florida, UNITED STATES

## Abstract

**Purpose:**

Optical coherence tomography angiography (OCT-A) enables visualization of retinal microcirculation. As a potential influence of mydriatic eye drops on retinal vessel density (VD) was proposed, the purpose of the present study was to investigate an influence of 5% phenylephrine and 0.5% tropicamide on macula and peripapillary VD.

**Methods:**

30 eyes of 30 healthy persons were measured by en face OCT-A (Spectralis OCT II, Heidelberg Engineering, Heidelberg). Scans of the macula (12 sectors, region of interest, ROI: 6.10 mm^2^) and peripapillary region (4 sectors, ROI: 2.67 mm^2^) were performed before (-) and 30 minutes after application of phenylephrine 5% and tropicamide 0.5% (+) eye drops (scan size was 8.41 mm^2^). Macula microcirculation was quantified in 3 retinal layers (superficial vascular plexus (SVP), deep capillary plexus (DCP), intermediate capillary plexus (ICP)). Data analysis was performed with the Erlangen-Angio-Tool.

**Results:**

(I) Mean VD was 33.03±2.3 (SVP), 23.53±2.9 (ICP) and 25.48±4.2 (DCP) before and 33.12±2.4 (SVP), 23.74±2.9 (ICP) and 25.82±4.0 (DCP) with mydriasis respectively. (II) Sectorial analysis: 30.63±2.9–34.45±2.9 (-) and 31.04±2.9–34.34±2.7 (+) in SVP; 22.61±2.9–24.93±3.2 (-) and 22.75±2.5–25.20±3.0 (+) in ICP; 24.56±4.7–26.45±3.4 (-) and 25.00±4.1–27.07±3.5 (+) in DCP. (III) Peripapillary region showed a mean VD of 31.82±3.8 before and 31.59±4.3 after mydriasis. Sectorial analysis of VD yielded a range of 31.04±4.1–32.65±3.8 (-) and 30.98±4.4–31.89±4.1 (+). (IV) Macula and peripapillary VD were not different before and after mydriasis (p>0.05).

**Conclusion:**

Pharmacologic mydriasis did not influence retinal microcirculation in macula and peripapillary region enabling OCT-A scans with enhanced imaging process and scan quality.

## Introduction

Optical coherence tomography angiography (OCT-A) is a new non-invasive imaging technique enabling visualization of retinal microcirculation in healthy and diseased.[1] As an extension of the standard structural OCT, OCT-A is based on temporal changes in reflection caused by intravascular moving blood cells. Implementation of an eye tracking technology prevents artefacts caused by eye movements. As a no risk alternative to fluorescein angiography (FFA), OCT-A enables a more differentiated understanding of various ocular diseases [2] without the use of intravenous injection of fluorescent contrast agents.

As the application of OCT-A is increasing, it is important to know potential influencing factors on retinal microcirculation in healthy eyes in order to transfer these findings to pathophysiological alterations. In clinical routine, mydriatic eye drops (e.g. tropicamide or phenylephrine) are essential for funduscopy and morphometric diagnostics. So far only few clinical studies have investigated the influence of mydriatic eye drops on retinal vessel blood flow and vessel density (VD) with incongruent data. Retinal capillary perfusion (RCF) was seen to be significantly decreased after application of 0.5% tropicamide in healthy eyes, measured with scanning laser doppler flowmetry (SLDF).[3] After application of phenylephrine eye drops blood velocity in the optical nerve head was observed to be reduced in animal (monkeys, rabbits) and healthy human eyes.[4,5] Blood flow of the central retinal artery seemed not to be influenced by phenylephrine in humans.[4,5] Contrary, no differences in vascular reactivity of the major retinal arterioles haven been observed after local application of 1% tropicamide, 1% cyclopentolate and the combination of 0.8% tropicamide and 5% phenylephrine, respectively.[6] The only study up to now, offering OCT-A data, showed no reduction of macula and peripapillary VD after local application of 0.5% tropicamide. However, combination of 0.5% tropicamide and 0.5% phenylephrine yielded a significantly reduced peripapillary, but not macular VD.[7]

Combination of local tropicamide and phenylephrine achieves a higher and longer lasting mydriasis than tropicamide or phenylephrine alone, enabling a sufficient basis for clinical settings.[8,9] Thus, the aim of the present study was to investigate a potential influence of 5% phenylephrine and 0.5% tropicamide on macula and peripapillary microvasculature measured with en face OCT-A of Heidelberg OCT II Spectralis in healthy eyes.

## Material and methods

### Participants

The present study was designed as a prospective control study. Thirty eyes of 30 normal subjects were included (16 male, 14 female). Mean age was 26.2±8 years with a range of 19–58 years (women: 25.9±8; men: 26.6±9). All participants received a complete standardized ophthalmologic examination including slit-lamp biomicroscopy, funduscopy and Goldmann applanation tonometry. The presence of any eye disease was an exclusion criterion. No previous ophthalmic surgery or laser treatment was performed. Intraocular pressure was to be within the normal range. The study protocol was approved by the local ethic committee of Erlangen and was performed in accordance with the tenets of the Declaration of Helsinki. Informed written consent, approved by the ethic committee of Erlangen, was obtained from all participants.

### Pupil dilation and OCT-A

A high resolution OCT-A of macula and peripapillary region was performed before and after local application of phenylephrine 5% and tropicamide 0.5% eye drops considering a respective residence time of at least 20 minutes. One eye of each subject was chosen randomly. En face OCT-A imaging was done by Heidelberg OCT Spectralis (Heidelberg, Germany). Scans of the macula and peripapillary region were performed by the same experienced investigator (M.M.). Imaging were recorded with a 15°x15° angle and a lateral resolution of 5.7 μm/pixel, resulting in a retinal section of 2.9 mm x 2.9 mm. TruTrack^®^ eye tracking and a projection artefact removal (PAR) algorithm were used in order to provide high transverse and axial resolution.

### Erlangen Angio-Tool (EA-Tool)

**Fig 1:**
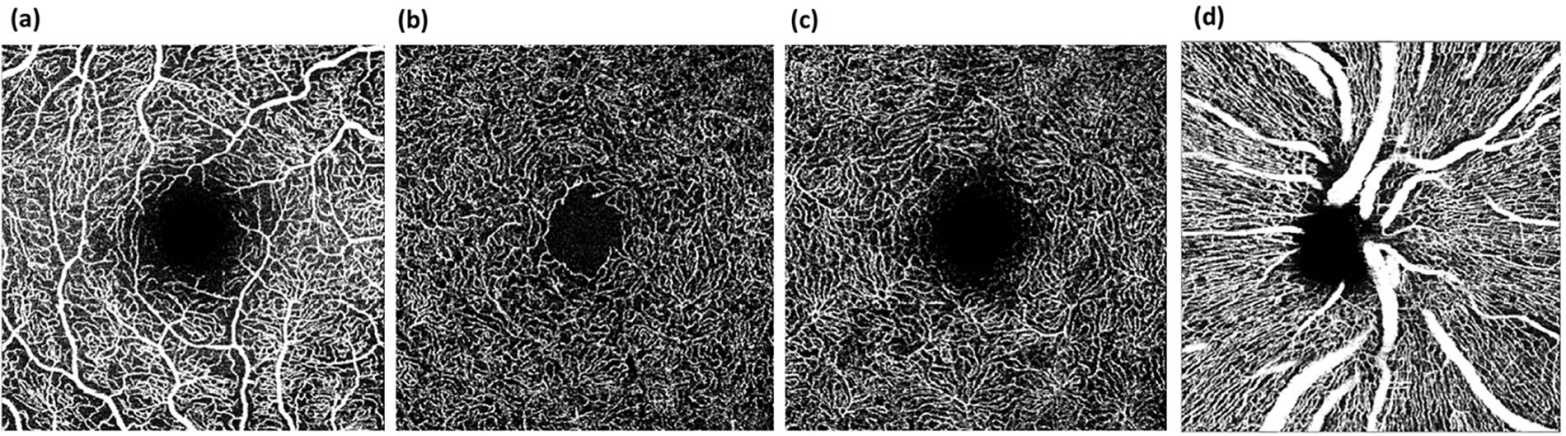
OCT-A images of the macula with superficial vascular plexus (SVP, a), intermediate capillary plexus (ICP, b) and deep capillary plexus (DCP, c) as well as peripapillar region (d).

The EA-Tool (version 1.0) implements multiple segmentations, enabling quantification of VD with a high level of reliability and reproducibility.[10] The software was coded in Matlab (The MathWorks, Inc., R2017b). Macula data of SVP, ICP, DCP, and of the peripapillary scan were scanned with a size of 2.9 mm x 2.9 mm (total scan size 8.41 mm^2^, Fig 1).

**Fig 2:**
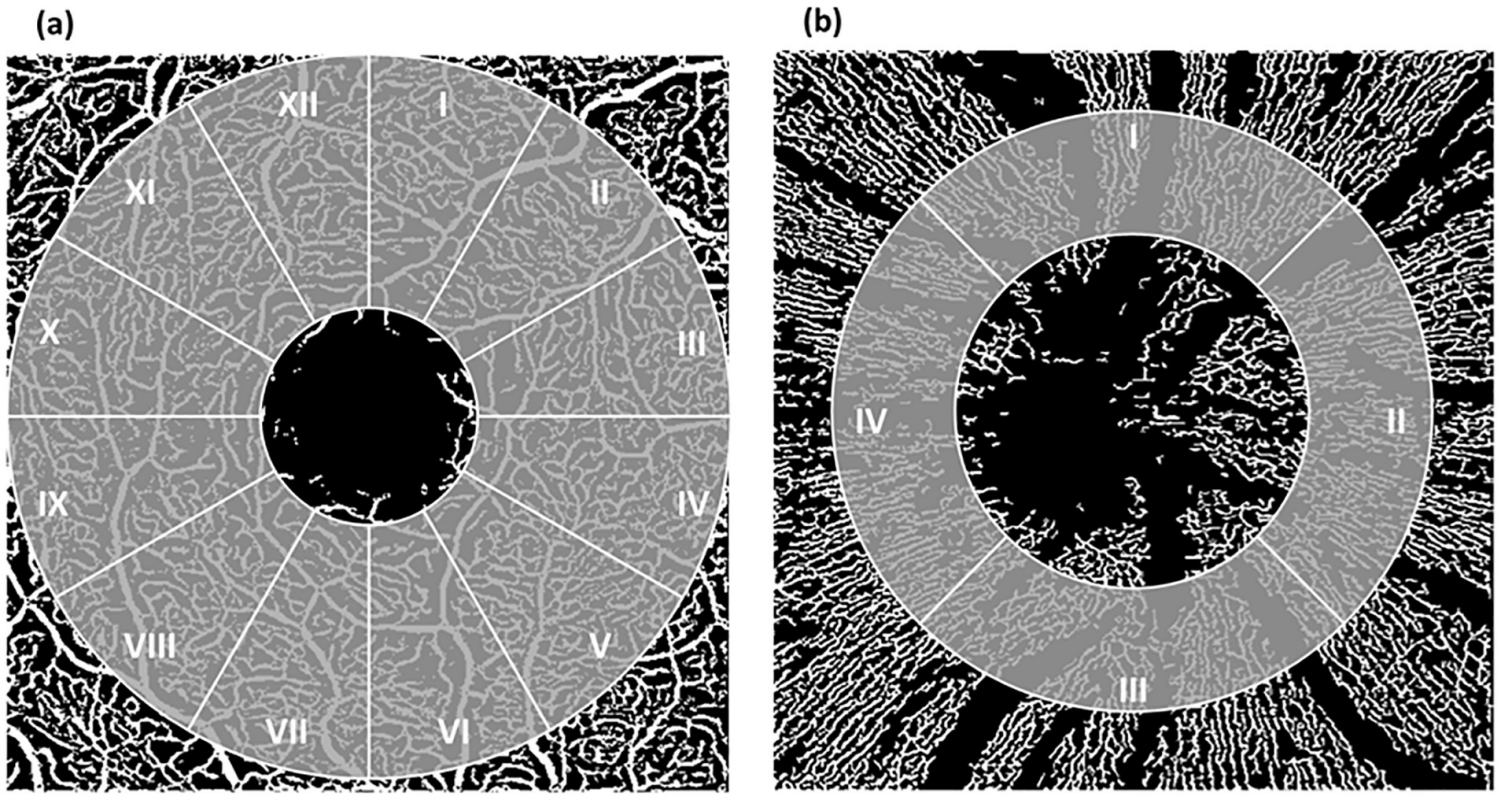
Region of interest of OCT-A scans analyzed with the Erlangen Angio Tool: Macula (subdivided into 12 sectors, a) and peripapillary region (subdivided into 4 sectors, b).

The data were exported into EA-Tool and analyzed separately. All scans were manually checked for shadows, artefacts and correct segmentations prior to any analysis. Overall and sectorial VD of the macula was analyzed. Analyzed region of the scan size was 6.10 mm^2^ with additional subdivision into 12 sectors for the macula region (á 30°, Fig 2a). Radius of the outer ring was 0.65 mm and inner ring (i.e. excluded FAZ) was 0.4 mm, in SVP, ICP, and DCP, respectively. Analyzed region of the peripapillary region was 2.67 mm^2^ (inner radius: 0.6 mm, outer radius: 0.5 mm, Fig 2b).

### Statistical analysis

Statistical analysis was performed by SPSS version 21.0. Demographic and VD data were presented as mean and standard deviation (SD). Subgroup analysis of VD was done using 12 sectors (s1-s12). Wilcoxon signed-rank tests were performed for VD of SVP, ICP, DCP, and the peripapillary VD, respectively. All data were corrected after Bonferroni considering multiple testing.

## Results

Mean macula VD before mydriasis were 33.03±2.3 (SVP), 23.53±2.9 (ICP) and 25.48±4.2 (DCP), respectively. Mean VD was 33.12±2.4 (SVP), 23.74±2.9 (ICP) and 25.82±4.0 (DCP) after mydriasis. Data of the sectorial analysis of VD (s1-s12) of SVP, ICP, and DCP before and after mydriasis can be seen in Table 1, respectively.

**Table 1:** Mean vessel density (VD), standard deviation (SD) and difference of VD before and after application of 5% phenylephrine and 0.5% tropicamide in superficial vascular plexus (SVP), intermediate capillary plexus (ICP), and deep capillary plexus (DCP) considering all 12 sectors.

	SVP			ICP			DCP		
	Mean VD±SD			Mean VD±SD			Mean VD±SD		
	before	after	Difference	before	after	difference	before	after	difference
	application of 5% phenylephrine and 0.5% tropicamide			application of 5% phenylephrine and 0.5% tropicamide			application of 5% phenylephrine and 0.5% tropicamide		
s1	33.40±2.9	33.43±3.6	-0.03	22.89±2.8	23.40±3.0	-0.51	25.00±4.4	26.27±4.6	-1.27
s2	32.68±3.5	33.32±3.0	-1.62	22.67±3.2	23.42±3.2	-0.75	25.30±3.8	26.34±3.3	-1.04
s3	32.27±3.1	32.85±3.2	-0.85	24.81±3.0	25.20±3.0	-0.39	26.45±3.4	27.07±3.5	-0.62
s4	33.10±3.1	33.30±3.0	-0.2	24.93±3.2	25.00±3.2	-0.07	27.10±4.1	26.42±4.2	0.68
s5	33.78±2.9	33.53±2.5	0.25	23.40±2.8	23.21±3.0	0.19	25.36±4.3	25.00±4.1	0.36
s6	34.45±2.9	34.34±2.7	0.11	23.35±2.4	23.26±2.4	0.09	26.05±4.4	25.54±4.2	0.51
s7	34.37±3.0	34.33±2.4	0.04	23.32±2.5	23.57±2.2	-0.25	25.42±4.1	25.56±3.6	-0.14
s8	33.81±2.2	33.75±2.5	0.06	23.27±2.5	23.30±2.0	-0.03	24.98±3.8	25.10±3.3	-0.12
s9	32.00±3.0	31.39±3.7	0.61	24.58±2.7	24.16±3.0	0.46	25.50±4.4	25.16±4.4	0.34
s10	30.63±2.9	31.04±2.9	-0.41	23.73±2.9	24.50±2.9	-0.77	24.56±4.7	25.36±4.4	-0.8
s11	32.10±2.9	32.53±2.8	-0.43	22.61±2.9	22.75±2.5	-0.14	24.67±4.3	25.60±4.0	-0.93
s12	33.72±2.5	33.68±2.9	0.04	22.84±3.3	23.14±3.0	-0.3	25.31±4.2	26.42±4.5	-1.1

Mean vessel density of the peripapillary region was 31.82±3.8 before mydriasis. Sectorial analysis yielded mean VD of 31.04±4.1 (s1), 32.01±3.9 (s2), 31.56±3.2 (s3), and 32.65±3.8 (s4).

No significant differences between overall macula VD before and after mydriatic eyedrops were observed in SVP (p>0.05), ICP (p>0.05), and DCP (p>0.05). Additionally, vessel density of all 12 sectors did not show any statistically differences before and after mydriasis (p>0.05).

Mean VD of the peripapillary region was 31.59±4.3 after mydriatic eye drops. VD of each sector s1-s4 yielded a range of 31.01±4.2–32.22±4.5 (before) and 30.98±4.4–31.89±4.1 (after mydriasis). VD of each sector can be seen in Table 2. No significant differences were seen in peripapillary VD after mydriasis compared to without (p>0.05).

**Table 2:** Mean vessel density (VD), standard deviation (SD) and difference of VD before and after application of 5% phenylephrine and 0.5% tropicamide in the peripapillary region considering all 4 sectors.

	Mean VD±SD		
	Before	after	difference
	application of 5% phenylephrine and 0.5% tropicamide		
	Mean VD±SD	Mean VD±SD	
s1	31.04±4.1	30.98±4.4	0.06
s2	32.01±3.9	31.69±3.8	0.32
s3	31.56±3.2	31.89±4.1	-0.33
s4	32.65±3.8	31.79±5.0	0.86

## Discussion

The novel technique of OCT-A promotes visualization and analysis of retinochoroidal microcirculation. As it is a non-invasive technique, retinal vessels density of the macula and peripapillary can be monitored *in vivo*. The lumen of a vessel is dynamic and regulated within a specific corridor. Veins are able to adapt to the blood flow (BF) demand by contraction and relaxation of pericytes. Arteries can act and react by smooth muscle contraction of the intima and media. It is important to know physiologically factors and drugs influencing retinal perfusion(e.g. age,[11] ethnicity [11,12] and exercise [13]) in order to distinguish these findings from pathological alterations. Yet, little is known about a potential mydriatic effect on retinal microcirculation and these data were not consistent until now. [3–7] Previous data of Scanning laser Doppler flowmetry (SLDF) provided evidence that retinal capillary perfusion (RCF) seemed to be influenced by local application of 0.5% tropicamide in normal subjects.[3] Additionally, first OCT-A data in normal eyes showed that peripapillary VD might be influenced by local application of phenylephrine 5% and tropicamide 0.5%. However, this effect was not observed for macula VD in healthy eyes, presenting only data of one overall macula layer.[7] Thus, the data of the present study aimed to investigate the effect of local application of phenylephrine 5% and tropicamide 0.5% on macula and peripapillary VD in normal healthy eyes, in a multi-layer evaluation. Macula VD was seen to be significantly different between all 3 retinochoroidal layers in normal subjects. Additionally, a slight effect of position might be seen in SVP. VD did not show any significant alterations in the macula and peripapillary region after mydriasis compared to without in normal eyes. Thus, phenylephrine 5% and tropicamide 0.5% did not show any effect on healthy retinochoroidal microcirculation.

En face OCT-A with Spectralis II OCT, a technical extension of the OCT, enables high resolution analysis of retinochoroidal microvasculature in all retinal layers with near to zero projection artifacts. This non-invasive imaging technique is based on motion contrast and consecutive changes within the signal-amplitude between two repetitive scans of the same retinal region. This technique in combination with the highly reliable semi-automated vessel density evaluation software (EA-Tool 1.0) enabled to detect even smallest changes in retinal microvasculature.[10] Scans with shadows, artefacts and segmentations were excluded in order to analyze scans with high quality. The ROIs were scanned with a high-resolution of 5.7 micrometer per pixel scan on a 2.9 x 2.9 mm OCT-A window, as a high resolution is necessary to detect even minor changes on a capillary level.

The effect of autonomic innervation of retinal vasculature is not total understood up to now. Only few clinical studies were available up to now, investigating the effect of anti-muscarinergic (e.g. tropicamide) and adrenergic (e.g. phenylephrine) agents.[3,6,7] Harazny et al. observed that RCF significantly decreased by 31.9 ± 13% after local application of 0.5% tropicamide in healthy eyes.[3] Peripapillary VD was seen to be reduced after local application of phenylephrine 5% and tropicamide 0.5%, yet not macula VD in healthy eyes, measured with OCT-A.[7] Contrary, retinal vascular reactivity measured with Canon Laser Blood Flowmeter, did not show any significant changes after local application of 0.8% tropicamide and 5% phenylephrine in young healthy subjects. Additionally, no differences in vascular reactivity were seen after eye drops containing 1% tropicamide or 1% cyclopentolate.[6] The data of the present study went along with the latter data of Tsui et al.[6] No significant changes of macula and peripapillary VD were observed after local application of phenylephrine 5% and tropicamide 0.5% in normal subjects. The data of the only previous study of Cheng et al. [7] cannot be compared directly to the present one, as the authors used a different OCT-A tool (Optovue, Inc. Fremont, CA, USA) and analysis software (RTVue-SR Avanti software, version 2.0.5.39). Additionally, the scanning window was different (6 x 6 mm (macula) and 4.5 x 4.5 mm (papilla)) and VD data were presented in one overall retinal layer. The authors stated no significant difference in overall macula VD, yet a significantly reduced peripapillary VD after combination of 0.5% tropicamide and 0.5% phenylephrine.[7] The differences to the present data might be seen in the different hard- and software as well as in the different scanning windows as mentioned above. Employing the projection artefact removal (PAR) in the present study enabled analysis of deeper retinal microvascular layers. This algorithm can remove nearly all projection artefacts and shadows out of the deeper layers of OCT-A scans with a consecutive clear and unaffected view on ICP and DCP. Analysis of VD of different microcirculation layers is important as OCT-A data correlated well with anatomical structures.[14] The results of OCT-A studies offering no significant different vessel densities before and after mydriasis should be interpreted with caution. This non-significance could also be to the small sample size of the study group or the method at all. Next to different scanning sizes of OCT-A (2.9 mm x 2.9 mm) and e.g. scanning laser doppler flowmetry (SLDF, 2.7 mm x 0.7 mm) the methodical background differs between the diverse studies. SLDF, using the Doppler frequency shift principle, measures blood flow velocity, wheras OCT-A visualizes structures with moved particles in the retina as vessels. OCT-A in a commercially avaible device (e.g. Heidelberg Spectralis II OCT-A) just visualizes perfused vessels in a certain velocity range without measuring altered perfusion itself as the alteration may be still in the flow range of imaging resolution. This may be the reason, why vessel density parameters are not changed by tropicamide in the present study. Future devices with color coded flow images may be able to show a possible effect of tropicamide on retinal capillaries in OCT-A images.

Similar to clinical studies, only few animal studies are available showing effects of autonomic innervation on retina blood vessels. Adrenergic innervations was seen in retinal layers, especially at boarder between inner nuclear and inner plexiform layers in monkey, rabbit, cat.[15] This symphathic innervation only reached retinal areas until the lamina cibrosa. Afterwards the nutrition and oxygenation of the adjacent and peripheral retina is thought to be maintained via autoregulation. [15–17] Up to now, there is no evidence that sympathic and parasympathic neurons directly act on retinal microvasculature.[17,18] Tropicamide and phenylephrine are mydriatic agents, commonly used in clinical settings to induce mydriasis. Tropicamide is a vasoactive drug, targeting and blocking cholinergic receptors (3-fold selective for M_4_).[19,20] The physiological agent acetylcholine (Ach) affects muscarinic receptors on pericytes, especially next to capillary bifurcations in adult rat retina.[21] Additionally, ACh is known to stimulate synthesis of nitric oxide (NO), a potential influence on retinal microcirculation might be hypothesized. This endothelium‐dependent dilatation is thought to be transferred via gap junctions between endothelial cells and smooth muscles.[22] The fact that the macula vessels lack smooth muscles [23,24] might be one reason that tropicamide did not show alterations in macula VD in the present study. The α1-adrenergic agonist phenylephrine acts vasoconstrictive. In animal models (e.g. monkey) arterioles of the optic nerve head were seen to be reduced after phenylephrine.[5] This vasoconstrictive effect was also observed in healthy young subjects.[4] Macula vessels were dominated by pericytes with α2-receptors,[23] whereas peripapillary vessels show α1-receptors.[25] Thus, pericytes of the macula cannot be influenced by phenylephrine with consequently untouched VD.

The present study is not without limitations. Only two consecutive OCT-A scans were measured, thus potential long-term effects of local phenylephrine 5% and tropicamide 0.5% might not be observed. A second limitation is the small OCT-A window covering a retinal area of 8.41 mm^2^. Yet, this ROI was chosen in order to scan with a high resolution. The present study was done in healthy persons. Within this study population neither an increase nor a decrease of macula and peripapillary VD were seen after local phenylephrine 5% and tropicamide 0.5%. However, differences in VD might be observed in ocular diseases with impaired vascular autoregulation. Thus, further studies are necessary to investigate VD in different ocular and vascular diseases.

## Conclusion

Local application of phenylephrine 5% and tropicamide 0.5% did not influence retinal microcirculation in macula (SVP, ICP, and DCP) and peripapillary region in healthy eyes. Thus, OCT-A scans can be done after mydriasis in order to increase the quality of the OCT-A scans and imaging process.
